# Chronic Uncontrolled Hypertension in the ED—We Hold This Truth to Be Self Evident

**DOI:** 10.1111/acem.70409

**Published:** 2026-09-11

**Authors:** Nicholas Eric Harrison, Phillip Levy

**Affiliations:** ^1^ Indiana University School of Medicine Indianapolis Indiana USA; ^2^ Wayne State University School of Medicine Detroit Michigan USA

In this issue of *Academic Emergency Medicine*, Akhetuamhen et al. describe practice pattern variation in management of patients with uncontrolled chronic hypertension, stratified by their emergency department (ED) reason for visit (RFV). Their findings largely reflect what one may expect intuitively: when hypertensive ED patients listed hypertension as their primary RFV, they were more than twice as likely to receive a prescription for an antihypertensive medication at discharge. Yet overall, regardless of RFV, antihypertensive prescribing rates were abysmal at 46.6% for those who came to the ED specifically asking for help managing their hypertension and just 21.2% for patients with another RFV who had uncontrolled blood pressure (BP). Importantly, because uncontrolled hypertension was defined as systolic BP ≥ 160 mmHg and/or diastolic BP ≥ 100 mmHg at triage and upon discharge, rather than < 130/80 mmHg which is the current American Heart Association (AHA) guideline [1] recommended definition of control, a goal achieved in only 20% of hypertensive patients nationwide, these numbers unfortunately and undoubtedly overrepresent the practice of antihypertensive prescribing writ large.

Sadly, these findings are not surprising and may even be validating to some as management of uncontrolled chronic hypertension has long been seen as beyond the purview of emergency physicians. However, evaluation and management of hypertension, and the ED's role in doing so, have changed markedly over the past 2 decades. In 2025 the American College of Emergency Physicians (ACEP) recommended, for the first time, that ED clinicians consider prescribing antihypertensives for appropriate asymptomatic patients at the time of ED discharge [2]. This reflected a major departure from prior ACEP policies on the topic, which generally discouraged chronic hypertension management in the ED. The paradigm shift in ACEP's policy responds to the abundance of evidence showing that patients may have the ED as their first or only touchpoint for management of hypertension [2]. The AHA also recently addressed the ED's role in hypertension management, highlighting what many ED clinicians will likely identify intuitively: an ED visit represents a unique opportunity to identify and treat those patients who have fallen through the cracks in the American healthcare system, among whom racial minorities and economically marginalized patients predominate [1].

While these results should be taken with caution given the study's many limitations including reliance on retrospective data, potential inaccuracy of RFV coding in the National Hospital Ambulatory Medical Care Survey (NHAMCS), and use of a cohort that was treated (from 2016 to 2022) prior to both the ACEP and AHA guideline updates, antihypertensive therapy is the cornerstone of BP control and the work of Akhetuamhen et al. stands to highlight the missed opportunity for public health benefit that could be derived from a more proactive mindset when it comes to the millions of patients who present to the ED with uncontrolled chronic hypertension [3, 4]. Prior work by Brody et al. supports the safety and efficacy of such an approach, with significantly lower systolic BP at outpatient follow‐up and no difference in adverse events for patients who were discharged from the ED with an antihypertensive prescription [5]. More recent data extend these observations, with lower rates of future hypertensive emergencies and fewer repeat ED visits among those with uncontrolled chronic hypertension who received an antihypertensive prescription at ED discharge [6]. Such information, along with the recent ACEP and AHA recommendations, should give comfort to emergency physicians who may be reluctant to prescribe BP lowering medications, knowing that this intervention will at worst do no harm, and at best help save a life down the road.

With respect to the analysis itself, one could quibble with the lack of a coherent conceptual model and adjustment for a wide range of downstream variables, many of which likely represent colliders and/or mediators rather than true confounders, creating the potential for spurious associations. But doing so would detract from the real contribution that Akhetuamhen et al. make to the literature, which is to affirm once again that a giant problem exists that is entirely of our own making. Increasingly, the weight of the evidence shows that emergency physicians can make a significant and lasting impact on ED patients with uncontrolled chronic hypertension, especially when paired with interventions that address gaps in access to care and other health disparities. This was shown in a recently published quality improvement project focused on linking vulnerable ED patients with uncontrolled hypertension to primary care via community health workers, where a 16 mmHg reduction in systolic BP was noted at follow‐up^4^. Randomized trials in the ED population, like the recent TOUCHED study, have also suggested a role for multi‐component interventions including pharmacy consultation and mobile‐health implemented home BP monitoring to reduce BP long term [7].

Clearly there is more work to be done. As professional organizations like ACEP and AHA recognize the low‐risk/high‐reward proposition of managing uncontrolled chronic hypertension identified in the ED, providers must come to see such encounters as an opportunity to impact public health and patient‐level outcomes, particularly for the most vulnerable individuals we care for. The low rates of prescribed antihypertensive therapy reported by Akhetuamhen et al. support a self‐evident but largely unacknowledged truth in our specialty—we can and should do our part when it comes to chronic hypertension care.

## Author Contributions

N.H. and P.L. contributed equally to all domains of the study.

## Funding

The authors have nothing to report.

## Disclosure

No large language model was used to write, prepare, or edit the current manuscript.

## Conflicts of Interest

The authors declare no conflicts of interest.

## Data Availability

Data sharing not applicable to this article as no datasets were generated or analysed during the current study.
